# DAMPs in Unilateral Ureteral Obstruction

**DOI:** 10.3389/fimmu.2020.581300

**Published:** 2020-10-07

**Authors:** Maja Wyczanska, Bärbel Lange-Sperandio

**Affiliations:** Department of Pediatrics, Dr. v. Hauner Children’s Hospital, University Hospital, Ludwig Maximilian University, Munich, Germany

**Keywords:** damage-associated molecular patterns (DAMPs), unilateral ureteral obstruction (UUO), inflammation, innate immunity, kidney

## Abstract

Damage-associated molecular patterns (DAMPs) are released from tubular and interstitial cells in the kidney after unilateral ureteral obstruction (UUO). DAMPs are recognized by pattern recognition receptors (PRRs), which mediate the initiation of an immune response and the release of inflammatory cytokines. The animal model of UUO is used for various purposes. UUO in adult mice serves as a model for accelerated renal fibrosis, which is a hallmark of progressive renal disease. UUO in adult mice enables to study cell death, inflammation, and extracellular matrix deposition in the kidney. Neonatal UUO is a model for congenital obstructive nephropathies. It studies inflammation, apoptosis, and interstitial fibrosis in the neonatal kidney, when nephrogenesis is still ongoing. Following UUO, several DAMPs as well as DAMP receptors are upregulated. In adult UUO, soluble uric acid is upregulated and activates the NOD-like receptor family, pyrin domain containing-3 (NLRP3) inflammasome, which promotes fibrosis, apoptosis, and reactive oxygen species (ROS) injury. Further DAMPs associated with UUO are uromodulin, members of the IL-1 family, and necrotic cell DNA, all of which promote sterile inflammation. In neonatal UUO, the receptor for advanced glycation endproducts (RAGE) is highly upregulated. RAGE is a ligand for several DAMPs, including high mobility group box 1 (HMGB1) and S100 proteins, which play an important role in renal fibrosis. Additionally, necroptosis is an important mechanism of cell death, besides apoptosis, in neonatal UUO. It is highly inflammatory due to release of cytokines and specific DAMPs. The release and recognition of DAMPs initiate sterile inflammation, which makes them good candidates to develop and improve diagnostic and therapeutic strategies in renal fibrosis and congenital obstructive nephropathies.

## Introduction

Sterile inflammation is a response to acute or chronic tissue injury without pathogens being involved. However, how does the body recognize damage? The activation of the immune system as a response to pathogens is possible by detection of molecular motifs conserved in so-called pathogen-associated molecular patterns (PAMPs). In the case of sterile injury, the immune system reacts in a similar way. Damage-associated molecular patterns (DAMPs) are intracellular molecules that are released as a response to sterile injury and are able to activate innate immunity just like PAMPs. DAMPs and PAMPs are recognized by pattern recognition receptors (PRRs), which then mediate the initiation of an immune response (1, 2). PRRs can be of several types, like Toll-like receptors (TLRs), NOD-like receptors (NLRs), AIM2-like receptors (ALRs), RIG-I-like RNA helicases, C-type lectin receptors (CLRs), and more (3, 4). DAMPs are molecules that have specific functions inside the cell; they operate as signals of cell damage only when they are released into the cytosol or the extracellular space (5). Released DAMPs expose hydrophobic portions of molecules that are naturally hidden within living cells and can thus be recognized as danger signals (6, 7). One way for DAMPs to leave the cell is to be passively released from dying cells. It is important to differentiate between different cell death pathways here. Apoptosis, being a non-inflammatory programmed way of cell death, does not lead to the release of DAMPs. By contrast, necroptosis, necrosis, and pyroptosis, induce inflammatory responses through the release of cytokines and DAMPs (8). DAMPs can also be secreted by living cells, which are exposed to life-threatening stress. High mobility group box 1 (HMGB1), for instance, is a DAMP that can be secreted by stressed cells without involving the endoplasmic reticulum (9). HMGB1 release is induced in monocytes by lipopolysaccharide (LPS), tumor necrosis factor (TNF)‐α or interleukin (IL)‐1. Upon activation, HMGB1 exits the nucleus into the cytoplasm, translocates into the cytoplasmic organelles and is released through lysosome exocytosis.

**Figure 1:**
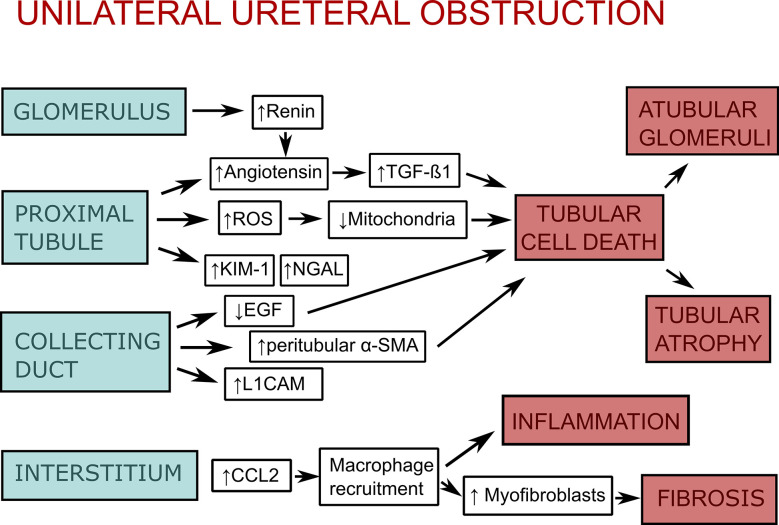
Scheme of the pathophysiology of unilateral ureteral obstruction (UUO). As a response to unilateral ureteral obstruction the glomerulus increases vascular renin production along with activation of the renin-angiotensin system, which leads to stimulation of transforming growth factor-β1 (TGF-1β). The proximal tubular epithelium activates the renin-angiotensin system as well. Additionally, it increases production of reactive oxygen species (ROS), which impair mitochondrial function, as well as kidney injury molecule-1 (KIM-1) and neutrophil gelatinase-associated lipocalin (NGAL). Collecting duct injury leads to downregulation of epidermal growth factor (EGF) and upregulation of peritubular mesenchymal collars that express α-smooth muscle actin (α-SMA) and L1 cell adhesion molecule (L1CAM). Injury of glomeruli, proximal tubule, and collecting duct lead to tubular cell death (apoptosis, necrosis, and necroptosis), which itself leads to atubular glomeruli and tubular atrophy. In the interstitium, there is an upregulation of chemokines (CCL-2, CCL-5) and adhesion molecules as an response to obstruction. This leads to macrophage recruitment, interstitial inflammation, and stimulation of myofibroblast proliferation, which causes fibrosis. The figure is adapted from (27).

**Figure 2:**
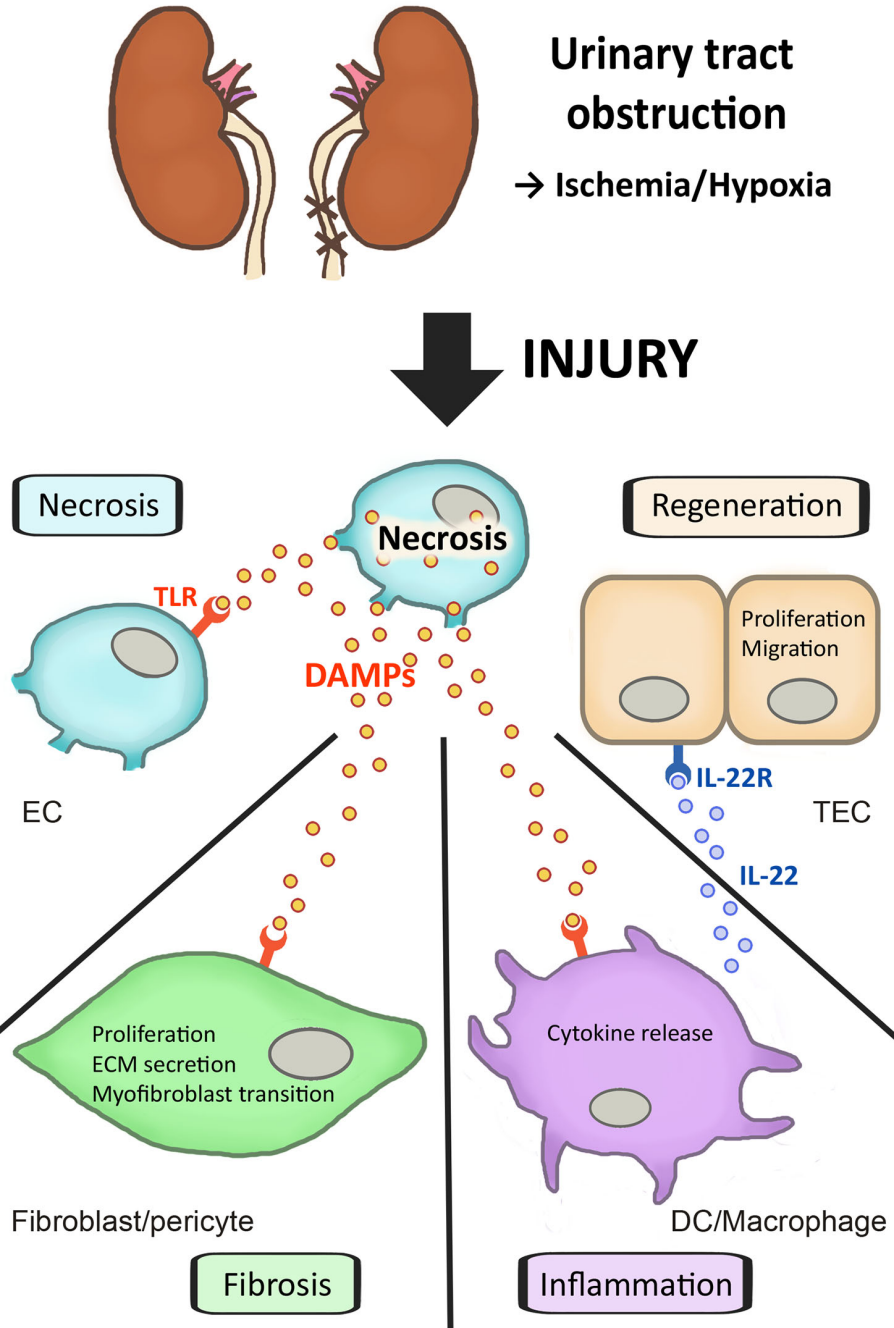
Different outcomes of cell death and DAMP release due to urinary tract obstruction. Unilateral ureteral obstruction causes cell injury and necrosis, as well as the regulated forms necroptosis and pyroptosis. Due to cell stress and cell death DAMPs are released by injured endothelial (EC) and tubular epithelial (TEC) cells. These DAMPs activate PRRs such as TLRs on other cells. This can lead to further renal cell necrosis, with amplification of DAMPs. Fibroblasts and pericytes activated by DAMPs trigger fibrosis through proliferation, ECM secretion and myofibroblast transition. Activated dendritic cells (DC) and macrophages release cytokines and chemokines, which initiate an inflammatory response. IL-22 secreted by renal DCs, on the other hand, is able to activate the IL-22 receptor on TECs, which accelerates tubular re-epithelialization, thus promoting regeneration of TECs.

DAMPs play a role in a variety of kidney diseases and could be used as biomarkers, or reveal novel drug targets for inhibiting the inflammatory response. In this review, we focus on DAMPs in unilateral ureteral obstruction (UUO) in mice (**Table 1**), which is used as a model for various purposes. UUO in adult mice is an experimental model of renal injury, leading to tubulointerstitial fibrosis (26). Renal fibrosis is the final common pathway of numerous kidney diseases leading to end-stage renal disease with dialysis or renal transplantation, as no effective treatments exits yet (23). UUO enables to study different stages of fibrosis development in an accelerated manner, like inflammatory cell infiltration, tubular cell death, extracellular matrix (ECM) deposition, and tubular atrophy (**Figure 1**) (28, 29). Urinary tract obstruction in the newborn mouse kidney also permanently impairs renal development (29). Neonatal UUO at the second day of life investigates the pathological mechanisms of congenital obstructive nephropathy (30, 31), which is the most common identifiable cause of chronic kidney disease in children and infants (32, 33). Neonatal UUO studies inflammation, apoptosis, and interstitial fibrosis in the neonatal kidney, while nephrogenesis is still ongoing. In humans, nephrogenesis is completed before birth by the 34-36 gestational week. By contrast, nephrogenesis in mice ceases 1-2 weeks postnatally. Therefore, performing UUO in newborn mice allows studying the effect of ureteral obstruction on kidney development. This experimental urinary tract obstruction in neonatal mice is analogous to the obstruction arising in the midtrimester human fetus with congenital obstructive nephropathy (28). UUO in neonatal and adult mice leads to sterile inflammation and thus to upregulation and release of DAMPs (**Figure 2**). DAMPs released during tissue injury, together with the immune receptors that recognize these, most likely contribute to the development of renal fibrosis (23). This review focuses on danger signals associated with obstruction in adult and neonatal kidneys.

**Table 1:** List of known DAMPs in UUO.

DAMPs	Putative Receptors	Pro-fibrotic	References
Biglycan	TLR2, TLR4, NLRP3	N/A	(10)
Decorin	TLR2, TLR4	⚬	(11, 12)
HMGB1	TLR2, TLR4, TLR9, RAGE	⦁	(13–15)
IL-1α	IL-1R	N/A	(16, 17)
IL-33	ST2	⦁	(18, 19)
Necrotic DNA	TLR9, ALR	⦁	(20, 21)
sUa	NLRP3	⦁	(22)
S100A8/A9	TLR2, TLR4, RAGE	⦁	(23, 24)
Uromodulin	TLR4	⚬	(25)

Black dot indicates that the criterion is fulfilled; white dot indicates that the criterion is not fulfilled; N/A, no data available for the UUO model. TLR, toll-like receptor; RAGE, receptor for advanced glycation end-products; NLRP3, NOD-, LRR-, and pyrin domains-containing protein 3; HMGB1, high mobility group box 1; ALR, absent in melanoma 2-like receptors; sUa, soluble uric acid.

## Fibrosis

Renal fibrosis is the hallmark of progressive renal disease and involves glomerular sclerosis and interstitial fibrosis (26). Fibrogenesis is considered a failed wound healing process after an injury. Processes leading to fibrosis are: proliferation of interstitial fibroblasts with myofibroblast transformation and deposition of a large amount of ECM components (34). There are several markers that are used to characterize fibrosis, like α-smooth muscle actin (αSMA) (34) or collagen I, III, and IV (35). Epithelial-mesenchymal transition (EMT) is also a process associated with UUO and renal fibrosis. It is a mechanism by which epithelial cells lose their cell polarity and cell-cell adhesion, and gain migratory and invasive properties to become mesenchymal stem cells (36). These multipotent stromal cells can differentiate into a variety of cell types. Epithelial cells dedifferentiate to mesenchymal cells as a repair mechanism, however, in the case of chronic or repetitive injury they can differentiate into myofibroblasts, thus building fibrotic scar tissue (37, 38). Following UUO monocytes infiltrate the renal interstitium and release cytokines such as transforming growth factor β1 (TGF-β1) (26). TGF-β1 promotes either apoptosis of tubular epithelial cells, leading to tubular atrophy, or EMT, leading to fibrosis. Several DAMPs, like IL-33, HMGB1, and biglycan also play a role in EMT (36, 39–41). Chronic inflammation is a critical process in fibrogenesis. Following kidney injury various pro-inflammatory stimuli activate fibroblasts (42). Fibroblasts can be activated by DAMPs through ligation with TLRs (43) (**Figure 2**).

## Cell Death and Inflammasomes

Necrosis and apoptosis are cell death mechanisms and both occur during UUO (28, 44). During apoptosis the plasma membrane integrity is maintained, whereas during necrosis it raptures (45). Additional difference between apoptosis and necrosis is the release of DAMPs, which is absent in apoptosis. Necrosis was seen as an unregulated form of cell death for a long time, but there are several cell death modalities of regulated necrosis, like necroptosis and pyroptosis. Engagement of receptors for Fas, TNF, or TNF-related apoptosis-inducing ligand **(**TRAIL) can lead to programmed cell death, apoptosis, through recruitment and activation of caspase-8 (46). However, in presence of caspase-inhibitors the cell death mechanism switches to a more rapid and necrotic mode of cell death, so-called necroptosis (47). Necroptosis is a well-characterized form of regulated necrosis, mediated by receptor interacting protein kinase-3 (RIPK3) and its substrate mixed lineage kinase like (MLKL) (48). Necroptosis is seen as a trigger for inflammation through release of DAMPs due to rapid cell rupture (49). DAMPs released through cell death can themselves trigger other endothelial and epithelial cells to undergo further cell death (**Figure 2**). It is unlikely, that both necrosis and necroptosis release the exact same cluster of DAMPs. DAMPs released by accidental cell death and secondary necrotic cells are well studied, however, studies about specific DAMPs associated with necroptosis were rarely conducted until recently (49). A crucial step of the necroptotic cell death pathway is phosphorylation of MLKL, thus pMLKL is seen as a marker for necroptosis (50).

Pyroptosis is a programmed necrosis that involves the activation of inflammasomes (45). Inflammasomes are intracellular sensors, they can be activated by extracellular DAMPs through the ligation of DAMPs and TLRs on cell surface. This process activates the nuclear factor (NF)-κB signaling pathway (51) or mitochondrial ROS production (22) and triggers the inflammasome. NOD-like receptor family, pyrin domain containing-3 (NLRP3) is one of the best-studied inflammasomes. Its activation results in inflammation. NLRP3 canonical activation in macrophages and other immune cells requires two steps: priming and activation. Priming is stimulated by binding of DAMPs or PAMPs to TLRs or cytokine receptors. It generally involves NF-κB signaling and expression of NLRP3, pro-caspase-1, pro-IL-1β, and pro-IL-18 (52, 53). Activation induces inflammasome assembly and caspase activation. NLRP3 proteins bind to apoptosis-associated speck-like proteins, which recruit pro-caspase-1 proteins that are cleaved into mature caspase-1. Active caspase-1 then processes pro-IL-1β and pro-IL-18 into mature IL-1β and IL-18. It also cuts gasdermin-D into N-terminal and C-terminal fragments, of which gasdermin-D-N creates extensive membrane pores, causing leakage of IL-1β, IL-18, and other cell compartments, which sets up the sterile inflammation (53). This process is called pyroptosis. Known DAMPs released due to pyroptosis after NLRP3 activation are HMGB1, IL-1α, and apoptosis associated speck-like protein containing a CARD (caspase activation and recruitment domain) (8). These are also known to be upregulated after UUO (13, 54, 55). The NLRP3 inflammasome itself can be activated by a variety of DAMPs such as uric acid crystal, silica crystals, ATP, asbestos, alum, and X-ray (53).

## IL-1α and IL-33

IL-1α and IL-33, which count as cytokines as well as DAMPs, are actively released during necroptosis (16, 45). IL-1α can also be actively secreted by epidermal epithelial cells (56). IL-33 was detected in the plasma of RIPK1-deficient mice and is dependent on the presence of RIPK3 and MLKL (16). Taken together with the observations of increased IL-33 expression in necroptotic epidermal keratinocytes (57), IL-33 was categorized as a necroptotic DAMP. IL-33 is able to activate basophils and eosinophils, as well as to induce type 2 immune responses (58). Recently it was shown that necroptosis and necroinflammation are accompanied phenomena in neonatal kidneys with ureteral obstruction (17). Biochemical analyses showed a decrease in caspase-8 expression and an increase in RIPK3 and pMLKL expression, indicating a role of necroptosis in UUO. IL-1α and IL-33 were measured in this study. IL-1α was significantly upregulated in the kidneys following obstruction. In the neonatal UUO model, in contrast to the adult one (18), the expression of IL-33 was downregulated. It is unknown, why IL-33 expression decreased in this particular disease model despite clear indications of necroptosis. A possible explanation is that interferon gamma (IFNγ), an immunomodulatory cytokine that was also upregulated following UUO, is able to downregulate pro-fibrotic IL-33 under certain conditions (59). IL-33 has been linked to fibrosis (18, 19) and can induce EMT *in vitro* (19). In HK-2 cells IL-33 was able to promote the cellular motility and migration capabilities of these cells. IL-33 activates the p38 mitogen-activated protein kinase (MAPK) signalling pathway, which induces the EMT process (60). The more pro-inflammatory and less pro-fibrotic state after neonatal UUO could be linked to the IFNγ activity and IL-33 downregulation. Even though the expression of only two possible necroptotic DAMPs were measured in this study, it can be assumed that due to necroptosis, other DAMPs are released as well and contribute to further inflammation and/or fibrosis after ureteral obstruction in the kidney.

## IL-1–Extended Family

Two members of the IL-1 extended family, IL-1α and IL-33 are referred to as cytokines that also act as DAMPs (61). The IL-1 extended family consists of IL-1α, IL-1β, IL-18, IL-33, IL-36α, IL-36β, and IL-36γ. All members play different biological activities involved in innate immunity (62). A recently published hypothesis argues that all members of the extended IL-1 family function as DAMPs (63). IL-1α and IL-1β are released from dying cells due to necroptosis and pyroptosis. Additionally, IL-18 is released from pyroptotic cells and IL-33 from necroptotic cells. Three of these cytokines (IL-1α, IL-1β, IL-18) are known to be upregulated during UUO (64, 65), IL-33 is upregulated in adult UUO (18), but downregulated in neonatal UUO (17). However, do all these cytokines qualify as canonical DAMPs? The definition of DAMPs is not completely clear. They are often portrayed as molecules that would only be released upon cell death and through certain pathways would initiate an inflammatory response (66). Most candidate DAMPs are structurally divers molecules that do not share common mechanisms of action. The author argues that the IL-1 family cytokines are good DAMP candidates due to their ability to drive inflammation in sterile injury. They are activated (IL-1β, IL-18, IL-36α, IL-36β, IL-36γ) and released mostly in cell death processes (67). One important argument is that the IL-1 family cytokines signal via receptors that contain intracellular Toll/IL-1 receptor signaling motifs, just like a subset of PAMPs. Additionally both receptor classes share signaling intermediates, like MyD88, and IRAK kinases. The main difference is stated in a part of the definition of DAMPs. DAMPs have a non-inflammatory “day job” within the cytosol or nucleus of cells, which cytokines generally do not have (68). Most members of the IL-1 family (IL-1β, IL-18, IL-36α, IL-36β, IL-36γ) require proteolytic processing to activate their biological activity, putting them in an inactive state, without function, until activation of the inflammasome or necrosome. An exception here would be cellular IL-1α, which has been associated with cellular senescence and other functions, as well as IL-33, which is expressed by a variety of cells and has a possible role in regulating gene expression (69). Given this, only IL-1α and IL-33 can be seen as both cytokines and DAMPs. However, if the cellular non-inflammatory active function of DAMPs would be neglected, then the other cytokines could also be counted as DAMPs. The quiescent state of the IL-1 family cytokines inside the cell seems to be the only argument against their categorization as DAMPs.

## Soluble Uric Acid

Uric acid, a purine catabolism product, is a DAMP released from injured and dying cells (70). Upon crystallization, it activates the immune system. It triggers the NLRP3 inflammasome activation through phagocytosis (71). Recently it has been reported that beside uric acid crystals, soluble uric acid (sUA) is also able to act as a DAMP and activate the NLRP3 inflammasome (22). Elevated serum uric acid induces inflammation dependent on mitochondrial ROS production and changes in the redox state. It is released in a hypoxic environment (72) and able to trigger NLRP3 through production of mitochondrial ROS, which leads to caspase-1 activation and IL-1β production. To study this, a murine UUO model was used, as it leads to increased levels of sUA. Additionally, correlation between tissue damage and the degree of sUA formation was observed (22). This also confirms the findings that NLRP3 plays a role in the acute phase following UUO (51, 73). Accordingly, NLRP3^-/-^ mice demonstrate reduced inflammation, tubular injury and fibrosis after UUO, which is associated with reduced caspase-1 activation and IL-1β and IL-18 maturation (64, 65). Inhibition of NLRP3 in UUO would have beneficial effects, as it plays a key role in sterile inflammation and fibrosis. However, NLRP3 is also involved in antiviral responses (74), so it may be more beneficial to inhibit DAMPs, like crystalized or soluble uric acid that trigger this inflammasome, without losing the protection from viruses. Inhibitors of uric acid like the xanthine oxidase inhibitors allopurinol and febuxostat are used to treat chronic kidney disease patients with hyperuricemia (75). Allopurinol treatment in UUO mice reduces type 1 collagen mRNA levels and hydroxyproline, the main amino acid that forms collagen (22). It also reduces the mRNA expression of *Il-33* and *Nlrp3*. The administration of febuxostat to mice after UUO inhibits the induction of proinflammatory and fibrogenic cytokines (76). It suppresses TGF-β, type I collagen and α-SMA expression and thus fibrosis. Treatment of patients with obstructive nephropathies with allopurinol or febuxostat may be therefore promising in the suppression of uric acid induced fibrosis.

## Necrotic Cell DNA

DNA has the ability to impact immunity itself or by forming complexes with other molecules and create unique danger signals (20). For the stimulation of immunity DNA has to have access to internal cell sensors. Extracellular DNA, either released from pathogens or by necrosis of host cells, can reenter another cells when bound to antibodies or nucleic acid-binding proteins. Necrotic cell DNA triggers dendritic cells and macrophages to mature phenotypically and functionally (77). As UUO in mice induces tubular necrosis (44), necrotic DNA is released and functions as DAMP in this model. Absent in melanoma 2 (AIM2) inflammasome is typically activated by pathogen DNA and triggers innate immunity, but it can also be activated by DNA released from dying cells (78). AIM2 is a cytosolic PRR that assembles an inflammasome in response to double-stranded DNA. Its activation drives proteolytic maturation of the proinflammatory cytokines IL-1β and IL-18, and pyroptosis (78). AIM2 has a protective role in microbial infection but a pathological one in sterile inflammation. Recently it has been shown, that *Aim2* deficiency reduces renal injury, fibrosis, and inflammation in adult mice after UUO (21). AIM2 is upregulated in the tubular epithelium and in inflammatory infiltrates in the kidney. In UUO-induced renal inflammation and injury, AIM2 is activated in recruited macrophages by uptake of necrotic cell DNA and aggravates the pathological state. *Nlrp3*^-/-^*Aim2*^-/-^ mice were used to examine the relative contribution of the inflammasomes NLRP3 and AIM2 to renal injury. There were no significant phenotypic differences between *Nlrp3*^-/-^*Aim2*^-/-^ mice compared with *Aim2*^-/-^ mice, suggesting a partially redundant role for the inflammasomes during renal injury. *Nlrp3*^-/-^*Aim2*^-/-^ mice had less injury, inflammation, and fibrosis compared with WT mice. However, still ongoing injury and inflammation in the injured kidney indicates an important role of other inflammasomes after ureteral obstruction. It also shows that inhibition of just one type of inflammasome might be able to reduce sterile inflammation and fibrosis, but not prevent it entirely.

## Mitochondrial DAMPs

Mitochondrial dysfunction plays an important part in various chronic inflammatory diseases, including UUO (64, 79). Mitochondrial damage causes production of mitochondrial reactive oxygen species, aberrant calcium mobilization, potassium efflux, reduction in cytoplasmic levels of NAD^+^, and upregulation of extracellular ATP (80). These changes have been shown to be involved in NLRP3 activation. In case of mitochondria injury or dysfunction production of mitochondrial DAMPs is possible (5, 80). Cytochrome C is a small soluble electron carrier hemeprotein that transfers electrons from complex III to complex IV to facilitate cell energy production (81). It is released in apoptotic cell death to trigger non-inflammatory cell death processes. However, when translocated into the extracellular space, cytochrome C functions as a DAMP. Cardiolipin is a phospholipid of mitochondria and confined to it (82). Due to mitochondrial stress or dysfunction it can undergo oxidation and be released into the extracellular milieu as a DAMP (83). Cardiolipin can directly bind and activate NLRP3 (84). Mitochondrial N-formyl peptides, which are released upon injury, can bind to formyl peptides receptors on neutrophils, monocytes, and dendritic cells and activate these (80). Mitochondrial DNA also seems to act as a DAMP (85). Upon opening of the mitochondrial permeability transition pore fragments of mitochondrial DNA are released from mitochondria (86). If this mitochondrial DNA enters thy cytoplasm, extracellular space or circulation, it can engage multiple pattern-recognition receptors and trigger pro-inflammatory responses (85). There is an ongoing debate, whether mitochondrial DNA is a bona fide DAMP following necroptotic killing (8). Recent findings however suggest that extracellular intact mitochondria are released during necroptosis and indeed act as danger signals (87). The released mitochondria were determined to be intact, as they did not emit detectible amounts of mitochondrial DNA. These extracellular mitochondria activate cytokine production in macrophages and cell maturation of dendritic cells, which classifies them as DAMPs (87). There has been no research on blocking these DAMPs in UUO, nonetheless UUO causes mitochondrial stress and dysfunction (64, 79), as well as necroptosis (17). It is probable that mitochondrial DAMPs play an important role in sterile inflammation and renal fibrosis following UUO. UUO decreases nuclear factor erythroid 2-related factor 2 (Nrf-2) translocation and activity, which is accompanied with an increase of mitochondrial BCL2 associated X protein translocation and an increase of cytosolic cytochrome c release (88). Overexpression of Nrf-2 attenuates mitochondrial dysfunction and has anti-fibrotic effects in UUO (88, 89). It is unknown whether the anti-fibrotic effect results from Nrf-2 induced reduction of TGF-β expression and hydroxyproline level alone or whether the reduction of cytochrome c release might also play a role. Research on mitochondrial DAMPs during UUO might be important in the future.

## S100A8/A9

The calcium binding protein S100A8/A9 is a DAMP that activates the receptor for advanced glycation end-products (RAGE) (24). RAGE is a multiligand pattern recognition receptor linked to chronic inflammation (90, 91). RAGE binds and mediates the cellular response to a variety of DAMPs. It is expressed at low level under normal physiological conditions, but is highly upregulated in chronic inflammation due to the accumulation of various ligands. RAGE has been identified as a receptor directly mediating leukocyte recruitment *in vivo*. S100A8/A9 heterodimer is expressed and released by phagocytes and has been shown to induce chemotaxis, cytoskeleton reorganization, and cytokine expression through activation of macrophages and neutrophils (92). It can be either passively released via necrosis, cellular damage, or neutrophil extracellular traps formation of myeloid cells, or actively from myeloid cells during acute or chronic local inflammation. S100A8/A9 exerts a critical role in initiating an inflammatory response by stimulation leukocyte recruitment and inducing cytokine secretion (93). Adult S100A9^-/-^ mice lacking the S100A8/A9 heterodimer that were subjected to UUO were protected from renal fibrosis (23). S100A8/A9 mediates renal fibrosis, tubular apoptosis, and crucial events for epithelial-mesenchymal transition in the kidney after UUO. High concentrations of S100A8/A9 induce a caspase-independent cell death, possibly necrosis, in tubular epithelial cells, thus leading to further release of DAMPs. Blocking S100A8/A9 activity has been shown to be beneficial in a variety of diseases (92, 93) and it could be a crucial factor for the reduction of fibrosis. Furthermore, RAGE, the receptor of S100A8/A9 is upregulated early in neonatal mice after UUO (94). This upregulation induces activation of the transcription factor NF-κB and its target genes, including proinflammatory cytokines. RAGE^-/-^ mice showed less tubular apoptosis and less interstitial fibrosis after neonatal UUO (95). Besides inhibition of inflammasomes and specific DAMPs, blocking DAMP receptors may be a promising target to treat sterile inflammation and fibrosis.

## HMGB1

High-mobility group box-1 (HMGB1) is also shown to be a DAMP released by necroptotic cells (96). HMGB1 is the best characterized DAMP and it is also a ligand of RAGE. (97). It has been identified as an important extracellular mediator in local and systematic inflammation (98). In the nucleus, HMGB1 organizes nucleosomes and DNA and regulates gene transcription (99). Due to cell injury or activation, nuclear HMGB1 is released into the cytoplasm, where it is involved in inflammasome activation as well as regulation of the autophagy/apoptosis balance through activation of immune and endothelial cells. Translocated HMGB1 has chemokine, cytokine, neuroimmune, and metabolic activities (99). HMGB1 can be actively secreted by macrophages/monocytes in response to inflammatory stimuli or passively released by necrotic cells (49, 96, 100). The release mode of HMGB1 can be divided into two groups: burst-mode and sustained-mode. Different durations of the release, being either 7.1 or 109 min on average, were observed (96). In the burst-mode HMGB1 is rapidly released from the cytoplasm, probably due to existing cytoplasmic membrane damage, in the sustained-mode the release is slowed down. The sustained-mode release of HMGB1 shows a balance between the extent of pore forming activity and membrane repair capacity of associated proteins. The biological significance of these two different modes remains unclear. However, it demonstrates a possible plasticity of cell death pathways and release of DAMPs. Thus, HMGB1 could play a role in prognosis and therapy. Furthermore, an acidic environment is able to trigger HMGB1 release *in vitro* (13). Thus, besides cell death it is hypothesized that acidosis, due to UUO or other pathologies, may cause release of HMGB1 or other DAMPs leading to inflammation. HMGB1 is upregulated after UUO in adult mice (13–15). It can induce the classically activated macrophages (M1) phenotype at the early stage of UUO (13). M1 activation is associated with injury, inflammation, and production of reactive nitrogen and oxygen species. Inhibition of HMGB1 diminished the presence of M1 macrophages (13). The treatment also resulted in an upregulation of M2 macrophages in the early stage of injury. As no previous M2 macrophage activation was observed in this stage of UUO, the M2 macrophages after inhibition of HMGB1 were likely to be converted from M1 macrophages. Additionally, inhibition of HMGB1 attenuated UUO-induced interstitial inflammation and blocked the injury-induced collagen deposition in the kidney. This indicates an important role of HMGB1 in sterile inflammation and fibrosis after UUO. Another link to fibrosis is that HMGB1 expression is highly elevated in diabetic nephropathy, which results in apoptosis and EMT progression of podocytes due to inhibition of autophagy (39). Downregulation of HMGB1 inhibits EMT progression.

## Decorin and Biglycan

Decorin and biglycan are small leucine rich proteoglycans. They are important components of the ECM. Recent studies however also show their involvement in different signaling pathways, indicating a role in autophagy, host immune responses and fibrosis (11). Decorin is the best studied proteoglycan; it regulates collagen fibrillogenesis and is a key factor for the mechanical integrity of such tissues as skin, tendon and ligaments (101). Additionally, it interacts with a variety of growth factors and thus has tumor suppressive, anti-inflammatory and anti-fibrotic properties. Decorin can be cleaved by proteases and cytokines and the cleavage fragments act as DAMPs. Decorin and biglycan activate as DAMPs the production of TNF α, IL-12, and macrophage inflammatory protein 2 in macrophages by binding to TLR4/2 (11). Decorin has anti-fibrotic activities through inhibition of TGF-β activities (101). Furthermore, decorin inhibits connective tissue growth factor signaling in fibroblasts, inhibits apoptosis of renal tubular epithelial cells and down-regulates microRNA miR-21 (43). The inhibition of these processes further alleviates interstitial fibrosis. In UUO decorin is highly upregulated (12, 102). Furthermore, decorin deficient mice show aggravation of renal fibrosis, highlighting the anti-fibrotic properties of this proteoglycan (12). Inhibition of decorin in renal sterile inflammation would have negative effects on fibrosis. However, inhibition of cleaved decorin, which functions as a DAMP, or the factors necessary for the cleavage could have beneficial effects. This should be elaborated in future research.

Biglycan can be found in most tissues as a stationary component of the ECM (10). However, upon release from injured cells or secretion by activated macrophages, biglycan becomes available in its soluble form and acts as a DAMP. Biglycan is involved in the activation of the NLRP3 inflammasome in sterile inflammation, leading to secretion of mature IL-1β. Similar to decorin, the expression of biglycan is upregulated after UUO (12). Biglycan deficient mice after UUO display lower levels of active caspase-1 and mature IL-1β, leading to reduction of infiltrating macrophages and less renal injury (10). Inhibition of biglycan attenuates inflammation, but its role in renal fibrosis is not yet clear (11). However, an upregulation of biglycan induces EMT by TGFβ activation (36). Biglycan binds extracellular TGFβ1 and modulates its access to the TGFβ receptors. TGFβ induces EMT, via a group of specific transcription factors (36, 103). Hence, an upregulation of biglycan seems to have pro-fibrotic properties.

## Uromodulin

Uromodulin (UMOD), also known as Tamm-Horsfall protein, is the most abundant protein in normal human urine (104). UMOD has been assigned a role in a variety of functions: modulating renal ion channel activity, intertubular communication, salt/water balance, inflammatory response, mineral crystallization, and bacterial adhesion (105). UMOD is synthesized in thick ascending limb tubular epithelial cells (106). It reaches the plasma membrane in a monomeric form. Its luminal release into the urine and subsequent polymerization is dependent on its cleavage mediated by the serine protease hepsin (107). Additionally, small amounts of UMOD are also released basolaterally into the interstitium and blood and show a positive association with kidney function (108). However, this positive effect results from monomeric UMOD. Polymeric UMOD in serum stimulates an inflammatory response (109). Many studies do not distinguish between these two possible states of UMOD. Polymerized UMOD is not immunostimulatory inside the tubular lumen, but once leaked into the interstitial compartment, it functions as a DAMP (56). UMOD can activate TLR4 on myeloid dendritic cells, leading to maturation of these cells (110). It has also the ability to activate the NLRP3 inflammasome leading to IL-1β release (111). Recently, UUO studies with UMOD deficient adult mice were conducted (25). In UMOD^+/+^ mice UMOD protein expression increased 9-13x above sham levels following obstruction. In UMOD^-/-^ mice apoptosis and cellular debris were reduced. The intensity of the interstitial inflammatory response was evaluated by F4/80 monocyte/macrophage protein levels. These were significantly lower (50%) in the UMOD^-/-^ mice, showing a proinflammatory function of UMOD after UUO. However, there were no significant difference in renal fibrosis between UMOD^+/+^ and UMOD^-/-^ mice. This suggests that in the absence of UMOD interstitial macrophages are recruited that are distinct and functionally polarized to a more robust fibrogenic phenotype. Blocking extratubular polymerized UMOD may be an interesting target to treat patients with obstructive nephropathies.

## Discussion

Extensive progress has been made in the field of DAMPs in recent years. New DAMPs and the corresponding pathways have been identified, as well as different release modes. DAMPs play an important role in UUO, as they drive inflammation and can have pro-fibrotic functions. They present possibilities for new biomarkers and anti-inflammatory therapies. At present, there is a lack of precise and reliable markers of urinary tract obstruction (31). Prenatal diagnosis of obstructive nephropathies is important as it allows for the planning of appropriate prenatal and postnatal care. It is key to distinguish between kidneys that do not need surgery and kidneys that would deteriorate and lose function or growth potential without. The perfect biomarker for renal fibrosis should be specific, non-invasive, directly involved in the mechanisms of fibrosis, with the ability to reflect treatment effects, and have low or no background in healthy individuals (112). DAMPs secreted in the urine may be future biomarkers in patients with congenital obstructive nephropathies and renal fibrosis, respectively. In a variety of diseases DAMPs are already used as biomarkers (92, 93, 113, 114). They can differentiate between diseases (114) and recognize inflammation as well as the site of infection or sterile injury (92). DAMPs assign valid outcome prognoses (115, 116) and help to differentiate between beneficial and harmful immune responses (115). Recently it has been shown that different isoforms of HMGB1 provide information on the type of injury (113). HMGB1 can be either slowly excreted from stressed or inflammatory cells, or rapidly released from dying cells. Non-acetylated HMGB1 is released from dying cells, whereas acetylated HMGB1 is associated with active secretion. This finding improves diagnostics, as it helps to estimate the severity of the inflammatory response. As for diagnostic purposes it is advised to use a mix of biomarkers, as under specific circumstances one biomarker could be inhibited and thus deliver false results. This can be seen in the case of IL-33, which is used as a marker for necroptotic cell death. However, IL-33 wasn’t upregulated after obstruction in the neonatal kidney despite evidence for necroptosis (17). A variety of factors, especially organ development or different diseases can alter the expression of certain biomarkers. There is a risk of false negative or false positive results if not taken into account.

Besides being used as biomarkers, DAMPs and DAMP associated pathways may also play a role in therapy. Inhibition of cell death pathways, like necroptosis can be helpful in inflammatory diseases, but only if the necroptotic cell death plays a major role (117). Inhibition of inflammasomes and receptors can be beneficial in reducing inflammation and fibrosis in the kidney. Knock-out of *Nlrp3* and *Aim2* resulted in less injury, inflammation, and renal fibrosis after obstruction (21). Neonatal RAGE^-/-^ mice showed less tubular apoptosis and interstitial fibrosis after UUO (95). There are several inhibitors of NLRP3 or RAGE (55, 91), however inhibition of inflammasomes and their receptors can also be harmful, as this would hinder pathogen detection. PAMPs and DAMPs are both recognized by PRRs like TLRs (4, 118) Inhibition of receptors would also inhibit their ability to detect PAMPs and initiate an inflammatory response to fight the infection (118–120). If a sterile inflammation would be accompanied or followed by a bacterial infection, receptor inhibition would worsen rather than improve the state of the patient. Another important issue is that most inhibitors block only one inflammasome (55) and therefore reduce inflammation only to a certain extent. An alternative would be to block several DAMPs associated with sterile inflammation during UUO. Inhibition of HMGB1 attenuated UUO-induced interstitial inflammation and renal fibrosis (13). Adult S100A9^-/-^ mice that were subjected to UUO were protected from renal fibrosis (23). HMGB1 and S100A8/A9 are well studied DAMPs and a variety of inhibitors have been designed that are used to reduce inflammation in diseases and injury (92, 93, 113). These inhibitors are widely used against harmful inflammation; their use against fibrosis needs to be studied in future research.

It should be considered that DAMPs are not always harmful and can have beneficial effects on repair. TLR2 on renal progenitor cells is activated by certain DAMPs and accelerates tubular repair (43). Additionally, TLR4 on dendritic cells, when activated by DAMPs, triggers IL-22 release. IL-22 activates the IL22-receptor on tubular epithelial cells and accelerates tubular re-epithelialization (**Figure 2**) (43, 121). HMGB1 recruits bone marrow derived mesenchymal stem cells and thus promotes repair (6). It also plays a role in proliferation and differentiation of tissue-associated resident stem cells. Moreover, HMGB1 promotes angiogenesis, which is required for tissue repair. These differences between beneficial and harmful functions of HMGB1 may be due to its redox state (113, 122) and should be further investigated. Directly inhibiting DAMPs could be helpful to fight sterile inflammation, however fine tuning might be a better option. Overexpression of DAMPs is harmful; however, they also contribute to tissue repair and healing. Research into DAMPs as biomarkers and their use in therapeutic application, especially regarding inflammation and fibrosis in the kidney, is a promising field for future research. There are still many open research questions that need to be answered (**Table 2**).

**Table 2:** Open questions.

Do mitochondrial DAMPs contribute to renal fibrosis?
Do other inflammasomes, beside NLRP3 and AIM2, play a role in sterile inflammation and fibrosis after UUO?
Can DAMPs be used as suitable markers for renal fibrosis and the severity of UUO?
Are the results of DAMP inhibition seen in mice reproducible in patients?

## Author Contributions

MW and BL-S wrote the manuscript. All authors contributed to the article and approved the submitted version.

## Funding

BL-S is supported by a grant from the German Research Foundation (DFG La 1257/5-1).

## Conflict of Interest

The authors declare that the research was conducted in the absence of any commercial or financial relationships that could be construed as a potential conflict of interest.

The handling editor declared a shared affiliation, though no other collaboration, with the authors MW and BL-S.
